# The Effects of Urban Forms on the PM_2.5_ Concentration in China: A Hierarchical Multiscale Analysis

**DOI:** 10.3390/ijerph18073785

**Published:** 2021-04-05

**Authors:** Mingyue Jiang, Yizhen Wu, Zhijian Chang, Kaifang Shi

**Affiliations:** 1 School of Geographical Sciences, State Cultivation Base of Eco-Agriculture for Southwest Mountainous Land, Southwest University, Chongqing 400715, China; acebaby@email.swu.edu.cn (M.J.); wyz19981013@email.swu.edu.cn (Y.W.); pdszjchang@gmail.com (Z.C.); 2 Chongqing Jinfo Mountain Karst Ecosystem National Observation and Research Station, Southwest University, Chongqing 400715, China; 3 Chongqing Engineering Research Centre for Remote Sensing Big Data Application, School of Geographical Sciences, Southwest University, Chongqing 400715, China

**Keywords:** urban forms, PM_2.5_ concentration, modifiable areal unit problem, spatial heterogeneity, multiscale analysis

## Abstract

For a better environment and sustainable development of China, it is indispensable to unravel how urban forms (UF) affect the fine particulate matter (PM_2.5_) concentration. However, research in this area have not been updated consider multiscale and spatial heterogeneities, thus providing insufficient or incomplete results and analyses. In this study, UF at different scales were extracted and calculated from remote sensing land-use/cover data, and panel data models were then applied to analyze the connections between UF and PM_2.5_ concentration at the city and provincial scales. Our comparison and evaluation results showed that the PM_2.5_ concentration could be affected by the UF designations, with the largest patch index (LPI) and landscape shape index (LSI) the most influential at the provincial and city scales, respectively. The number of patches (NP) has a strong negative influence (−0.033) on the PM_2.5_ concentration at the provincial scale, but it was not statistically significant at the city scale. No significant impact of urban compactness on the PM_2.5_ concentration was found at the city scale. In terms of the eastern and central provinces, LPI imposed a weighty positive influence on PM_2.5_ concentration, but it did not exert a significant effect in the western provinces. In the western cities, if the urban layout were either irregular or scattered, exposure to high PM_2.5_ pollution levels would increase. This study reveals distinct ties of the different UF and PM_2.5_ concentration at the various scales and helps to determine the reasonable UF in different locations, aimed at reducing the PM_2.5_ concentration.

## 1. Introduction

With very large economic successes and a fast urban development since 2000 [1,2], air pollution problems have increasingly become an important issue in China [3]. The fine particulate matter (PM_2.5_) and other pollutants occurring in air have severely damaged human health in China [4,5,6]. Unfortunately, the impact of PM_2.5_ is widespread, as three out of four Chinese prefectures does not meet national ambient air quality standard limits, as reported by the China Environmental State Bulletin [7]. Additionally, high PM_2.5_ could lead to a poor visibility, thereby greatly affecting the appearance of cities and even increasing the possibility of traffic hazards. The aforementioned poor air quality has raised concerns across Chinese society, and all sides would like to address this issue and reduce the PM_2.5_ concentration.

So as to reduce the PM_2.5_ concentration, the first step should be to determine its driving factors. Source apportionment results have suggested that there are six common sources for PM_2.5_: vehicular emissions, soil dust, secondary aerosols, coal combustion, biomass burning, and industrial emissions [8]. Natural conditions could also generate origin-specific patterns of PM_2.5_ aggregation and dissipation, including the temperature, precipitation, humidity, air velocity, and terrain. More importantly, human activities therein have been proven to affect the concentration. Urban forms (UF), including urban sprawl, irregularity, compactness, quantified with relative urban form indexes, can reflect the spatial configuration of human activities, and represents the direction of urban development. The urban sprawl is calculated with four components: residential density, land use mix, street accessibility, and degree of centering [9]. The irregularity is calculated by the shape indexes, and the compactness is measured by urban aggregation. Seven metrics describing the urban sprawl (CA and LPI), irregularity (NP, LSI, and ENN_MN), and physical compactness (PLADJ and COHESION) have been applied to quantify the UF. By influencing land use, infrastructure, and resource allocation, UF can affect PM_2.5_ concentrations and pollution levels.

Since UF can play an essential part in controlling the PM_2.5_ concentration, many studies have quantified how specific UF affect the concentration from different perspectives [10,11,12,13,14,15,16]. To date, few studies have simultaneously considered the effects of spatial scale and heterogeneity on relationships between UF and PM_2.5_ concentration [17,18]. There exist three specific scientific research questions: (1) How do UF affect the concentration across Chinese urban area from a temporal perspective? (2) Would the relationships change with the scale across China? (3) What is the impact of spatial heterogeneity on the relationships?

In this study, the relationships between long time-series data of UF and PM_2.5_ concentration has been considered from a national perspective. Additionally, scale effects have been discussed. Urban system is a multiscale and spatially heterogeneous system, revealing a hierarchical structure of different centers or clusters across spatial scales [19]. For complex multiscale systems such as the Chinese urban system, the analysis scale usually affects the statistical analysis results, such as correlation and regression analysis of socioeconomic data [20,21]. Moreover, this study comprehensively analyzes the spatial heterogeneity effects. Spatial heterogeneity generally refers to uneven distribution of various landscapes and patterns within an area. Analyses have indicated that there are large differences between urban areas in China, as they exhibit spatial heterogeneity, including different development stages, spatial distribution non-equilibrium, regional heterogeneous pattern regularities, distinct disintegration, and inter-conglomeration heterogeneity [22]. The social, economic, and natural conditions in the different regions differ, and the relationships vary [23].

Therefore, our study explores these links across China from a multiscale perspective. The provincial scale and prefectural-level municipal (city) were selected as experimental objects. Abundant remote sensing satellite data was collected and assessed as PM_2.5_ and independent-factor data. We then employed land-use/cover data calculated from satellite-derived data to obtain a series of spatial metrics to quantify the UF. After the above data analysis, we deployed econometric regression models, i.e., panel data models, to explore the relationships between the UF and the PM_2.5_ concentration, on the basis of considering scale effects and spatial heterogeneity.

## 2. Materials and Methods

### 2.1. Study Area

**Figure 1:**
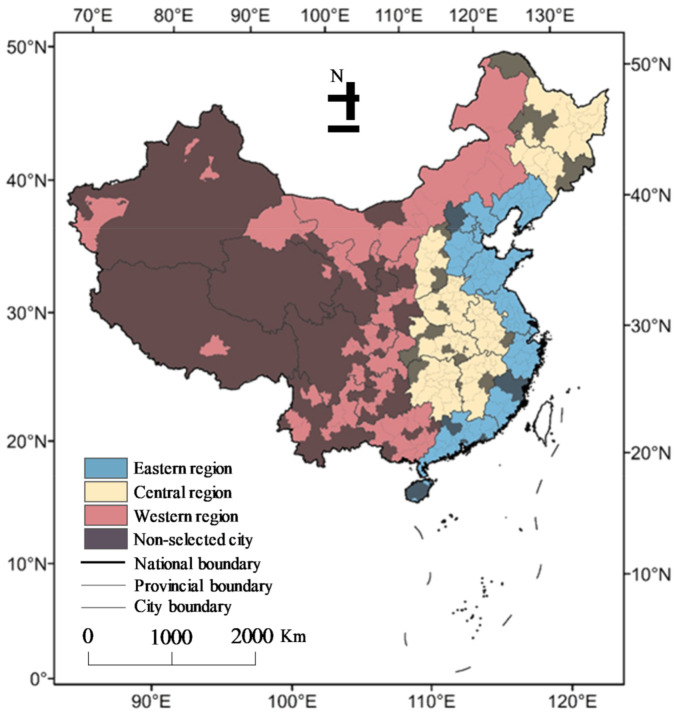
The distribution of the studied provinces and cities in China.

Twenty-seven provinces and 250 cities in China were chosen for this study (Figure 1). The selected provinces and cities are capable of supporting the research on scale effects and spatial heterogeneity and involve many typical natural characteristics. Urban planning has been conducted at provincial and city scales from a macro-perspective. Therefore, these two scales are the most basic administrative and spatial scales to study the relationship between Chinese urban areas and environment. In addition, in this study, three regions were divided, namely, the eastern (ER), central (CR), and western regions (WR), using the division method prescribed by the National Bureau of Statistics, according to the corresponding economic development levels and geographical locations. The ER refer to those provinces and cities with the earliest coastal open policy, and they are relatively economically well-developed areas. The CR pertain to the economically underdeveloped central areas, and the WR relate to the economically underdeveloped western areas. Regional division is helpful during heterogeneity analysis, as the relationships are investigated with different scales at various economic development levels and geographical locations. All of the cities and provinces were established before 2000 and exhibited varying degrees of development from 2000 to 2015. UF depends on the natural landscapes. In general, the inland flat areas have smooth urban area edges and a high urban cohesion.

### 2.2. UF Quantification 

Landscape metrics were calculated to represent the UF, as they have a good ability to explain the gaps between urban development and land-use approaches and heterogeneous urban landscapes [24,25]. Specifically, seven metrics were employed in this study (Table 1) as follows: (1) CA is the total urban area; (2) NP is the number of patches; (3) LPI is the largest patch index; (4) LSI is the landscape shape index; (5) ENN_MN is the average minimum adjacency distance; (6) PLADJ is the percentage of like adjacencies; and (7) COHESION is the patch cohesion index. Formulas for calculating these metrics are detailed in [16], and these metrics have been found to have impacts on PM_2.5_ pollution.

**Figure 2:**
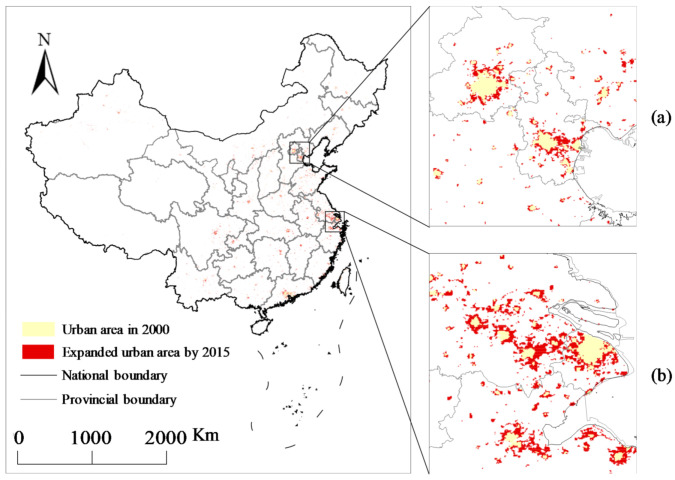
The dynamics of urban expansion in China from 2000 to 2015, especially in Beijing–Tianjin–Hebei (**a**) and Yangtze river delta (**b**) city clusters.

These seven metrics describe the urban sprawl (CA and LPI), irregularity (NP, LSI, and ENN_MN), and physical compactness (PLADJ and COHESION) [17]. Specifically, CA has continued to increase from 2000 to 2015 in China (Figure 2). Regarding the urban irregularity, LPI represents the largest patch percentage in the urban areas. NP measures the total number of urban patches, LSI measures the landscape area perimeter, and ENN_MN represents the spatial distance between any two closest neighboring patches. These three metrics investigate the urban land degree of fragmentation or subdivision, and they are proportional to the complexity of the city shape. Regarding the physical compactness, PLADJ measures the absolute degree of urban land aggregation, while the physical connectivity of urban areas is measured by COHESION, which is negatively correlated with the aggregate distribution of UF.

This study employed urban land datasets to quantify and represent UF. The stratified support vector machine (SVM) classification method was chosen (based on [26]) as a reference, the stratified support vector machine (SVM) classification method was chosen to measure the urban expansion at a 1-km spatial resolution using nighttime stable light (NSL), land surface temperature (LST) and normalized difference vegetation index (NDVI) data. Figure 2 shows the calculation results, demonstrating the urban expansion phenomenon in China from 2000 to 2015 and the spatial distribution of the land-use changes in the Beijing–Tianjin–Hebei and Yangtze River delta city clusters. After extracting urban built-up area, our selected metrics were computed by the above urban land datasets. Finally, we used “FRAGSTATS” software [27] to calculate the landscape metrics for each province and each city for each year separately as explanatory variables.

### 2.3. PM_2.5_ Data

The PM_2.5_ data were produced by the Atmospheric Composition Analysis Group and were obtained from their website (https://sites.wustl.edu/acag/datasets/surface-pm2-5/, accessed 18 August 2020). The datasets were gridded at the finest resolution of the information sources that were incorporated (0.01° × 0.01°), but do not fully resolve PM_2.5_ gradients at the gridded resolution due to influence by information sources at coarser resolution. The data extraction method involved estimating the ground-level PM_2.5_ total and compositional mass concentrations across China. To estimate, it considered the aerosol optical depth (AOD) retrieved from NASA Moderate Resolution Imaging Spectroradiometer (MODIS), Multi-angle Imaging Spectroradiometer (MISR), and Sea-viewing Wide Field-of-view Sensor (SeaWIFS) instruments with the GEOS-Chem chemical transport model. Subsequently, it calibrated the results to regional ground-based observations in terms of both the PM_2.5_ total and compositional mass concentrations via geographically weighted regression (GWR) as previously detailed [28]. The dataset was established then with combined geophysical-statistical estimates of the PM_2.5_, based on the geophysical satellite-derived values of van Donkelaar et al. [29]. They combined three satellite-derived PM_2.5_ sources to produce global PM_2.5_ estimates. For each source, they developed transport model to represent local aerosol optical properties and vertical profiles. The dataset can be used in global scale with a high accuracy [30], so we have selected the data from 2000 to 2015 in this study.

### 2.4. Control Variables

**Figure 3:**
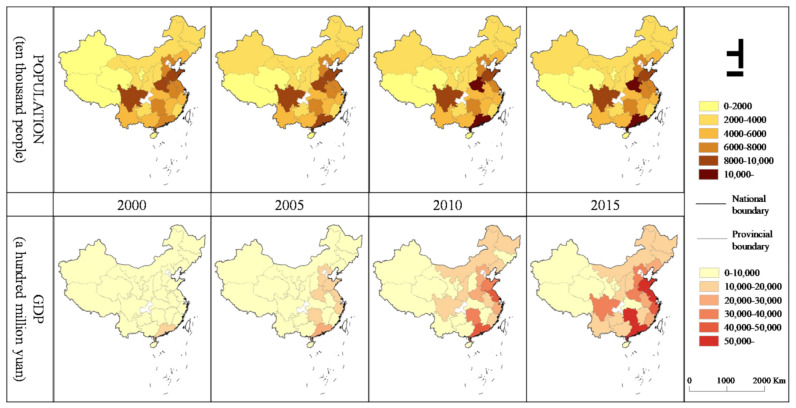
Spatial distribution of the GDP and population at the provincial scale.

**Figure 4:**
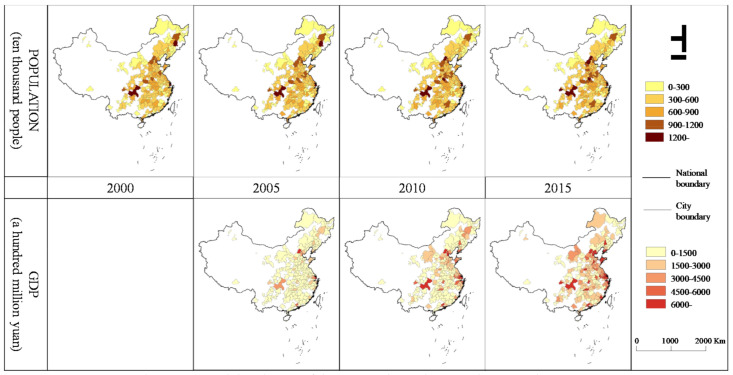
Spatial distribution of the GDP and population at the city scale.

The population and gross domestic product (GDP) were adopted as control variables. We collected urban population and GDP data from the China Statistical Yearbook, which shares the same time range as that of the PM_2.5_ and UF data, according to the administrative divisions (Figure 3 and Figure 4). These factors could influence both the PM_2.5_ concentration and UF. The urban population size was chosen as a control variable because it has been demonstrated to contribute to the PM_2.5_ concentration in China, exhibiting an inverse U-type relationship [31]. Additionally, local economic growth is another driving force impacting the PM_2.5_ concentration [32]. Shi, et al. [33] found that over the 15 years between 2000 and 2015, the speed and scale of urban space expansion in China far exceeded those of urban economic expansion. Only by controlling all the variables other than the independent variables triggering variations in the dependent variable can the cause-and-effect relationship be clarified in experiments, so adding these two variables was vital to facilitate the exploration of the relationships.

### 2.5. Econometric Model

A panel data model tracks the same set of individuals over time. Since panel data models possess both cross-sectional (n-dimensional individuals) and time dimensions (T periods), they have been widely applied as econometric models and therefore chosen in this study. In this research, the period from 2000 to 2015 was selected for the model. Before employing any panel data model, natural logarithmic transformation was applied to all the dependent and independent variables, thus avoiding any adverse effects due to “non-stationarity and heteroscedasticity. In addition, the study used panel unit root tests [34] and panel cointegration tests [35] to examine for stationarity and collinearity and avert false regression. Thus, the model was adjusted to:

(1) $$ln{\left(P\right)}_{it}=\alpha_{0}+ln(Z_{it})\delta +\varepsilon_{it}$$

where $P_{it}$ represents average PM_2.5_ concentration of year t in city or province i; $\alpha_{0}$ is the intercept; $Z_{it}$ represents a vector of the exogenous variables, which includes the seven UF metrics and control variables for each studied area unit in year t; δ is the coefficients of interest vector; and ε is the random error. Panel data models could be classified as fixed- and random-effect models. The formal model and assumptions are that, specifically, when $\alpha_{0}$ and $ln{\left(Z\right)}_{it}$ are related, the model is defined as a fixed-effect model. Conversely, it is referred to as a random-effect model. In this study, the model was applied at both the urban and provincial scales, and both fixed- and random-effect [36], which yielded different results.

## 3. Results and Discussion

### 3.1. Spatiotemporal Analysis of the UF and PM_2.5_ Concentration

The Chinese urban areas greatly increased between 2000 and 2015 (Table 1). At the provincial scale, we found that the urban area nearly tripled, with the maximum value 1.7 times and the minimum value 11 times larger than that in 2000. It is worth noting that the standard deviation of CA increased, which indicates that the regional development imbalance worsened. Along with urban growth, the urban area became increasingly irregular, and the links between cities tightened. COHESION and population both increased from 2000 to 2010 and decreased from 2010 to 2015, reflecting the tendency of the urban physical density to first increase and then decrease, probably due to the urban reconstruction rate decreasing with increasing new urban construction rate [37]. The population and GDP of the urban areas increased, and the GDP growth rate matches that of the population. [38]

Over the 15 years since 2000, dramatic changes in the urban landscape patterns in China have occurred (Table 2). The table indicates that there was a significant increase in CA and NP in selected cities, as the values increased several times due to the rapid economic development and urban sprawl. LPI recorded an increase in edge expansion, with urban infilling occurring as well, as the villages between cities and in certain small rural areas along city outskirts changed into urban areas, thereby exhibiting leapfrog development. The upward LSI trend suggests the rise in the spatial heterogeneity and irregularity of urban landscapes. In 2000, the average ENN_MN value was 27,942, and this value was nearly halved in 2015, indicating more connected urban patches, which could also be related to land-use changes. The PLADJ and COHESION values rose slightly but declined from 2010 to 2015, indicating that the urban areas became less concentrated or compact and experienced fluctuations.

**Figure 5:**
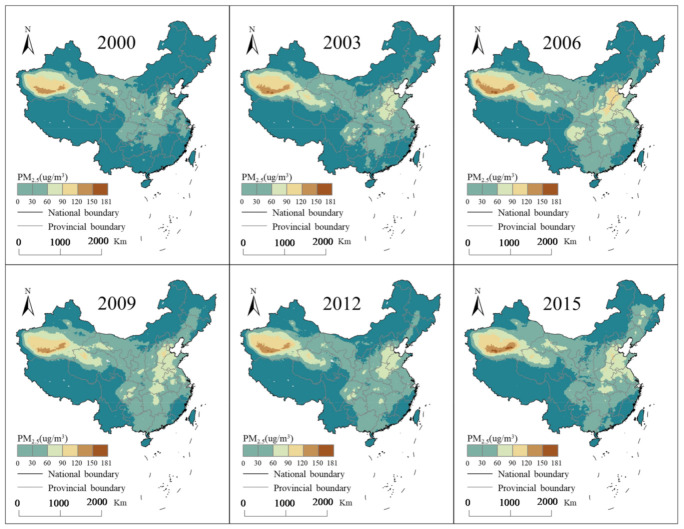
Spatial distribution of the fine particulate matter (PM_2.5_) concentration from 2000 to 2015.

China experienced a considerable rise in terms of the PM_2.5_ concentration, from 39.547 μg/m^3^ in 2000 to 49.613 μg/m^3^ in 2015, in addition to different spatial and temporal distributions with various UF, population sizes, and economic structures as well. However, an inflection point was observed because the data increased from 2000 to 2010 and decreased thereafter up to 2015, with Table 3 presents some statistical values of PM_2.5_ at provincial and city scales. Specifically, the maximum PM_2.5_ concentration rapidly decreased, indicating that in certain heavily polluted cities, pollution was effectively reduced. This phenomenon was obvious in North China, as shown in Figure 5. Most cities in North China have developed secondary industries and a high energy combustion level, resulting in high pollutant emissions. In addition, due to space-dependent effects, the PM_2.5_ pollution transported from neighboring provinces also resulted in high PM_2.5_ concentration. Another heavily polluted area is Northwest China, where the Taklimakan Desert lies, which has a dry climate that could easily cause dust storms or sandstorms. The figure shows a trend whereby the pollution on the right side of the Hu Huanyong Line mainly involves changes in the pollutant concentration. The Hu Line, also known as the “Hu Huanyong Line”, is an imaginary line stretching from Heihe (a northern city of China located on the Russian border) to Tengchong (a southwestern city of China bordering with Myanmar), which divides the area of China into two roughly equal parts [39]. Additionally, many studies have summarized the regular pattern of the PM_2.5_ concentration distribution across China. There are four areas with a high PM_2.5_ concentration, including the Huang-Huai-Hai Plain (eastern China), the Lower Yangtze River Delta Plain (eastern China), the Sichuan Basin (southwest China), and the Taklimakan Desert (northwest China) [40]. More specifically, Yan, et al. [41] found that in Hebei Province, the cities farther away from Bohai Bay exhibited higher PM_2.5_ concentration. Instead, cities along the coastline presented lower concentrations. Cities from the west to the Hu Line to the south to the Yangtze River revealed the highest PM_2.5_ concentration [42]. Thus, spatial heterogeneity analysis at different scales is also necessary in this study.

Due to the spatial heterogeneity of the PM_2.5_ distributions, the concentration within a province tends to remain similar, and the concentration difference between provinces is quite large, while the standard deviation of provincial-scale statistics exceeds that of city-scale statistics. All provinces in the study area were selected, and more cities were chosen in the eastern and central regions, while the western region exhibited a better air quality than the central and eastern regions. Hence, the average PM_2.5_ concentration at the provincial scale was higher than that at the city scale.

### 3.2. Correlations between the UF and PM_2.5_ Concentration in China

Unit root tests were performed first (Table 4). The test results indicated that the metrics were stationary at the first-order difference, and the nonstationary null hypothesis was rejected at the 1% significance level. Therefore, we could model the panel data based on the above results. In the study, fixed- and random-effect models were then employed for estimation.

**Figure 6:**
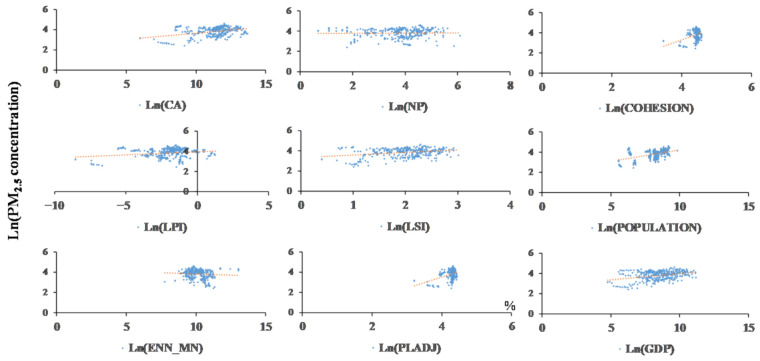
Scatterplots of urban forms (UF) metrics vs. PM_2.5_ concentration at the provincial scale.

**Figure 7:**
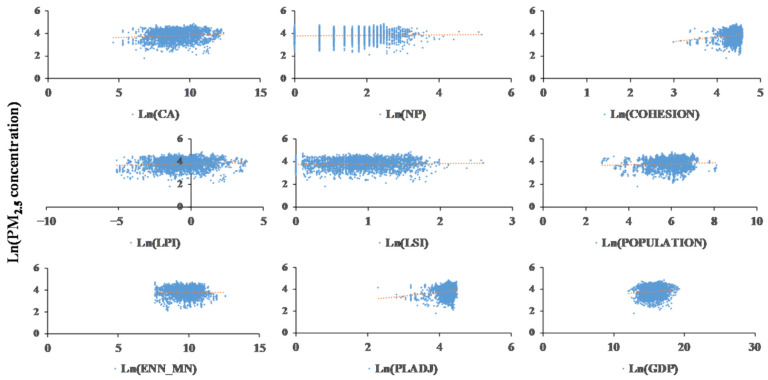
Scatterplots of UF metrics vs. PM_2.5_ concentration at the city scale.

Table 5 lists panel data coefficients calculated at the different scales and with the various models. As indicated by the table, the indicators noticeably correlated with the PM_2.5_ concentration accounted for the majority of the indicators. In order to show their relationships more clearly, Figure 6 and Figure 7 illustrate the scatterplots of the metrics and PM_2.5_ concentration.

At the provincial scale, CA imposed a noticeably positive effect on the concentration. LPI negatively correlated with PM_2.5_ concentration, and it was the most important factor under provincial-scale circumstances (a coefficient of −15.85). In terms of the urban irregularity, ENN_MN, LSI, and NP all showed negative impacts, as the diffusion of pollutants occurred more readily along the longer boundaries between urban and nonurban areas. In regard to the urban compactness, PLADJ exerted no significant effects. In addition, the COHESION statistics differed between the models and were significant at the 5% level. The control variables, including population and GDP, did not significantly influence the PM_2.5_ concentration at the provincial scale in the fixed effect model. Human activities differ among the various UF, and artificial urban planning determines the sprawl, irregularity, and compactness of cities. Human activities are usually accompanied by the consumption of energy, which directly leads to the emission of pollutants. In addition, the GDP reflects human activities as well. The prosperity of the industrial economy, which consumes significant energy, may highly aggravate the air cleaning burden. [44,45]

The variables affecting the PM_2.5_ concentration at the city scale were roughly the same as those at the provincial scale. CA had significant positive effects, and the effects were negative in terms of LPI, LSI, and ENN_MN. It is worth noting that no significant correlations were found between the urban physical compactness (PLADJ and COHESION) and the PM_2.5_ concentration with either model, indicating that the compactness or dispersion of urban buildings had little impact on the PM_2.5_ concentration. Among all variables, LSI was the most important factor at the city scale (a coefficient of −2.29) since cities were insensitive to UF irregularities.

### 3.3. Scale Effect of the UF on the PM_2.5_ Concentration

The results in Table 5 showed that the connections between the UF and PM_2.5_ concentration could differ depending on the scale, and findings were obtained by considering scale effects. First, the R^2^ values are greater at the provincial scale than at the city scale, proving that the conclusions are more credible at the provincial scale. Findings at the city scale may be promiscuous due to the diversity of cities. Overall, NP exerted a prominent negative effect on the PM_2.5_ concentration at the provincial scale. By contrast, hardly any significant correlations were found at the city scale. Regarding the urban areas at the provincial scale, a higher distribution of urban patch represented a more scattered or irregular urban pattern, leading to higher PM_2.5_ concentration. Second, LPI and CA imposed the most significant effects on the provincial- and city-scale PM_2.5_ concentration, negative and positive, respectively. The development of core cities could centralize resources by providing more jobs, high-quality education, and medical resources [46]. Third, the urban compactness showed various correlations with the PM_2.5_ concentration. Studies have found that it would be beneficial to reduce PM_2.5_ concentration to develop urban expansion in a continuous and aggregated pattern, which would increase transportation accessibility [47]. However, these effects were only observed at the provincial scale in this study and varied in the different models. Thus, detailed studies on the impacts caused by traffic are required considering different models.

### 3.4. Heterogeneity Analysis at the Different Scales

The urbanization process in China revealed significant regional differences, as the urban development speed in the eastern regions (ER) was higher than that in the central (CR) and western regions (WR) as shown in (Figure 3 and Figure 4), and the PM_2.5_ concentration showed spatial variations (Figure 5). This led to the following questions in this study: Is there a regional difference in the impact of the UF on the PM_2.5_ concentration? To answer this question, this study separated the ER, CR, and WR of China with the division method prescribed by the National Bureau of Statistics. This study then estimated the relationships between the UF and PM_2.5_ concentration across the ER, CR and WR at the provincial and city scales.

Table 6 provides the UF–PM_2.5_ concentration relationships across the different regions at the provincial scale and different estimation methods. Each index had a different influence on the results in the different regions. Generally, the ER and CR exhibited similar modes of influence, and the PM_2.5_ concentration was significantly influenced by all seven indexes except ENN_MN. Urban sprawl, irregularity, and physical compactness proved to be important in the above relationships in the eastern and CR. The results in the ER and CR were similar, due to both natural environmental reasons such as topography and meteorology [48] and, to a large extent, regional coordinated development. Because of their early and fast development, they had established regional linkages and economic interdependence. The similarity among industries resulted in similar impact mechanisms of the UF on the PM_2.5_ concentration. Notably, among the control variables, the GDP of the eastern provinces significantly impacted the PM_2.5_ concentration, while in the CR, the population was the significant influencing factor. Therefore, it is believed that the main factor influencing the concentration in the ER is economic development, and economic development negatively impacted the PM_2.5_ concentration. In particular, the tertiary industry in the ER of China has been vigorously developed in recent years, and the leading provinces have performed well in terms of industrial upgrading and transformation and have gradually eliminated energy-consuming industries emitting excessive pollution. However, in the CR, the population index had a significant impact on the concentration of pollutants, which indicates that the PM_2.5_ emissions caused by the large population in the CR cannot be ignored [49]. The CR lacks advantageous industries, which are mostly traditional industries and consume much manpower and resources. Focusing on completing the industrial upgrading and transformation in this region and promoting energy-saving measures may achieve the goal of reducing the PM_2.5_ concentration.

In the provinces in the WR, only three indexes imposed a significant impact on the above relationship. In the WR, the main factors influencing the observed relationships are natural factors, as the western provinces are characterized by an undulating topography, containing mostly mountainous and plateau areas, so the trend in the urban areas is greatly restricted by natural conditions [50]. Table 6 reveals that NP exerted a significant negative effect on the PM_2.5_ concentration, while ENN_MN had a significant positive effect on the PM_2.5_ concentration. The western provinces have a relatively small urban population. In the WR, both the population and GDP impacted the PM_2.5_ concentration.

Table 7 summarizes the estimation results of the UF–PM_2.5_ concentration relationships in the three geographical regions at the city scale and two estimation methods. As shown for the eastern cities, the indexes of urban sprawl (CA and LPI) and irregularity (NP, LSI, and ENN_MN) exerted a significant impact on the PM_2.5_ concentration. The irregular UF with lower NP values in the eastern cities produced higher PM_2.5_ emissions than those in the other regions. This result suggested that the scattered form played a vital part in the PM_2.5_ concentration in the northern region and slightly affected the concentration in the other regions. This indicates that the cities in the eastern region with a large NP value and long edges effectively reduced the concentration of pollutants. Certain cities in the east depend on the sea, and their irregular coastlines led to higher LSI values, and the diffusion of pollutants in the atmosphere–ocean cycle also helped reduce their concentrations in these cities. PLADJ and COHESION imposed no significant influence on the PM_2.5_ concentration, which may be due to the rapid development of the cities in the ER and convenient transportation services, so that the transfer of urban economic activities was less affected by the urban compactness. In the cities on the central plains with flat terrains, without any mountains and seas nearby, all the metrics had significant impacts on the PM_2.5_ concentration. The cities with larger urban areas, smaller patch numbers and largest patch areas, shorter patch lengths, fewer spatial connections between patches, higher proportions of similar patches, and less agglomeration exhibited an increase in the PM_2.5_ concentration. The PM_2.5_ concentration in the western region was affected by all metrics except PLADJ. It is worth mentioning that NP was significant in the western cities, indicating that among the provinces, scattered patterns played an important part in the PM_2.5_ pollution in the western cities.

### 3.5. Limitations and Future Directions

The following limitations are considered worthy of future researches in this study. First, the main source of the PM_2.5_ concentration in China is usually point source pollution, such as industrial discharge points. The locations of these activities are usually determined by human factors and less so by the UF, which could restrict the relationship between the UF and PM_2.5_ concentration. Second, the accuracy of the data is important to the final results. However, PM_2.5_ concentration data are obtained through remote sensing or station monitoring, with drawbacks of a low resolution or poor representation. Future research should therefore focus on PM_2.5_ concentration of detailed households, transport, and commercial buildings. In addition, the spatial distribution of residential, commercial, and industrial areas is crucial to the observed relationships, but at a resolution of 1 km^2^ it is almost impossible to determine very detailed land use from existing land use/cover datasets. Therefore, more detailed urban land use data should be derived and calculated. To better validate the models, model-observation comparison of the five-year change (i.e., Y2000 vs. Y2005, Y2005 vs. Y2010, etc.) would increase the persuasion, and special cases can be applied in future studies. Third, the UF affects the PM_2.5_ concentration through urban patches with various geometries. Therefore, only seven indicative metrics are not enough to reflect all UF, and future research should focus on selecting typical UF to modeling closer to the real phenomenon. Fourth, despite the panel data models captured the magnitude of the relationships [47], the causal relationships between them cannot be directly determined [51]. Fifth, the spatial dependence and heterogeneity of the UF and PM_2.5_ concentration are important factors influencing the above relationship. Therefore, simulation methods could be applied to characterize these inherent mechanisms. Sixth, only the population and GDP were adopted as control variables, while other factors that may influence the UF and PM_2.5_ concentration were not considered, which may lead to neglecting important influencing factors. For instance, the number of roads and the shape of their extension affects the UF and may further affect the concentration of PM_2.5_. Therefore, more control variables should be selected for comparative analysis in the future, which could enhance the study of the influence mechanism of UF–PM_2.5_ concentration relationships. Finally, the study would use some uncertainty analysis to reach a refined study of PM_2.5_ currents and diffusions inside the urban [52].

## 4. Conclusions and Policy Implications

The study has explored the relationships between the UF and PM_2.5_ concentration in China from a multiscale perspective (provincial and city scales) in 27 provinces and 250 cities. By integrating two control variables (the population and GDP), this paper establishes a comprehensive evaluation index system of UF based on the seven metrics describing UF. Specific panel data models were then applied to calculate the relationships between the UF and PM_2.5_ concentration. Subsequently, six conclusions are reasoned out, which are summarized as follows. First, the cities with high PM_2.5_ concentration s in China are mainly located in the eastern region and northwest desert area. Second, the effects of the UF–PM_2.5_ concentration relationship are attributed to LPI and LSI at the provincial and city scales, respectively. Third, NP imposes valid active effects on the PM_2.5_ concentration at the provincial scale, but it is not statistically significant at the city scale. Fourth, little correlation is found between the urban compactness and PM_2.5_ concentration at the city scale. Fifth, LPI exerts a significant positive effect on the PM_2.5_ concentration in the eastern and central provinces but imposes no significant effect in the western provinces. Sixth, in the western cities, the PM_2.5_ concentration increase is likely caused by the scattered and irregular urban pattern.

The above findings demonstrate the relationships between UF and PM_2.5_ concentration differently by listing the results at the different scales and in different regions. Rational urban planning based on local conditions by optimizing the existing urban spatial layout may be useful in achieving a reduction in PM_2.5_ concentration in China. Therefore, the research results can provide relevant references for urban planners. First, cities may rationally plan their expansion and optimize their scale across China, increasing urban green land. Second, consideration can be given to pooling resources and actively building core cities, especially in the eastern and central provinces. Third, industrial cities can choose to disperse factories with high pollutant emissions, so that cities and green spaces can interpenetrate each other and thus try to reduce PM_2.5_ concentration. Fourth, traffic construction in the western provinces may be accelerated to make the traffic flows between cities more convenient and promote the development of urban industries. Fifth, in the process of the Great Western Development Strategy, western cities could properly concentrate on the development of certain plots to control the number of possible patches in cities.

## Figures and Tables

**Table 1:** Statistics of the metrics at the provincial scale.

Year	STA	CA (km^2^)	NP	LPI (%)	LSI	ENN_MN	PLADJ (%)	COHESION	*Population (a)*	*GDP (b)*
2000	MIN	400.000	2.000	0.000	1.500	5000.000	25.000	32.827	259.830	117.800
	MAX	539,400.000	113.000	2.014	11.670	470,196.946	84.409	97.314	9488.000	10,741.250
	MEAN	82,784.615	32.346	0.181	5.735	56,061.794	73.487	82.181	4363.937	3389.905
	STD	104,858.622	26.361	0.376	2.617	86,408.089	11.103	11.128	2560.109	2810.902
2005	MIN	2000.000	3.000	0.001	2.188	10,909.704	37.500	51.233	280.310	248.800
	MAX	716,300.000	148.000	2.650	14.879	469,413.836	83.666	97.564	9768.000	22,557.370
	MEAN	141,515.385	54.385	0.296	7.849	46,840.972	73.505	84.960	4515.563	6655.292
	STD	152,436.225	36.338	0.513	3.256	85,780.566	9.020	8.343	2683.922	5949.370
2010	MIN	3200.000	5.000	0.001	2.667	9472.759	50.000	62.002	300.220	507.460
	MAX	781,900.000	161.000	3.352	16.237	126,236.657	83.745	98.068	10,440.940	46,036.250
	MEAN	175,407.692	63.308	0.387	8.737	30,699.052	74.230	86.224	4674.063	14,773.905
	STD	173,214.016	42.025	0.672	3.558	23,359.325	7.211	7.037	2845.617	13,010.691
2015	MIN	4500.000	12.000	0.001	3.643	6628.607	43.333	57.727	323.970	1026.390
	MAX	894,100.000	444.000	3.548	20.360	45,108.049	82.502	97.652	10,849.000	72,812.550
	MEAN	239,873.077	159.385	0.447	12.313	15,191.780	69.440	83.358	4794.344	24,381.921
	STD	198,278.027	99.308	0.718	4.092	8163.476	8.578	8.193	2930.681	21,446.614

Notes: STA indicates the statistic; MIN indicates the minimum; MAX indicates the maximum; MEAN indicates the average; STD indicates the standard deviation; a, indicates ten thousand people; b, indicates ten thousand yuan; data are missing in 2011, 2013 and 2014.

**Table 2:** Statistics of the metrics at the city scale.

Year	STA	CA (km^2^)	NP	LPI (%)	LSI	ENN_MN	PLADJ (%)	COHESION	*Population (a)*	*GDP (b)*
2000	MIN	1.000	1.000	0.006	1.000	2000.000	0.000	0.000	15.960	179,307.000
	MAX	1341.000	16.000	30.122	4.691	159,708.973	91.406	97.870	3091.090	45,511,500.000
	MEAN	99.282	3.489	1.156	2.002	27,942.287	67.689	75.962	437.640	4,132,103.206
	STD	178.799	2.800	3.329	0.824	26,968.348	15.390	15.033	333.963	5,072,992.580
2005	MIN	3.000	1.000	0.008	1.000	2000.000	27.778	35.317	17.220	695,200.000
	MAX	1990.000	24.000	37.932	6.151	159,708.973	89.088	98.679	3169.160	91,541,800.000
	MEAN	167.179	5.830	1.741	2.660	23,666.228	70.455	80.578	452.159	7,883,789.645
	STD	267.914	4.541	4.473	1.100	19,797.264	10.614	10.279	342.462	10,589,234.331
2010	MIN	7.000	1.000	0.008	1.000	2000.000	27.778	34.051	21.800	1,632,777.000
	MAX	2120.000	32.000	44.203	7.281	159,708.973	88.797	98.916	3303.450	171,659,800.000
	MEAN	205.614	6.646	2.110	2.935	21,795.357	71.442	82.124	466.032	17,404,371.670
	STD	310.517	5.134	5.244	1.190	18,472.615	9.526	9.429	333.169	21,837,487.314
2015	MIN	11.000	2.000	0.008	1.500	2000.000	31.250	48.759	20.250	1,900,441.000
	MAX	6498.100	2909.000	48.280	62.865	85,519.693	89.818	98.756	3371.840	251,234,500.000
	MEAN	561.008	27.610	2.467	4.090	14,475.278	69.050	81.098	478.639	28,409,807.169
	STD	433.857	194.195	5.727	4.227	11,252.983	9.700	9.170	343.803	35,861,986.988

Notes: STA indicates the statistic; MIN indicates the minimum; MAX indicates the maximum; MEAN indicates the average; STD indicates the standard deviation; a, indicates ten thousand people; b, indicates ten thousand yuan; data are missing in 2011, 2013 and 2014.

**Table 3:** Statistics of the dependent variables at different scales.

Year	STA	PM_2.5_ (μg·m^−^^3^) at the Provincial Scale	PM_2.5_ (μg·m^−^^3^) at the City Scale
2000	MIN	10.972	6.000
	MAX	75.282	92.607
	MEAN	40.253	39.547
	STD	15.885	17.999
2005	MIN	14.353	12.500
	MAX	81.991	99.800
	MEAN	50.792	50.620
	STD	17.303	18.455
2010	MIN	13.462	11.000
	MAX	85.721	123.773
	MEAN	52.393	52.994
	STD	18.742	20.170
2015	MIN	12.455	12.500
	MAX	80.981	92.900
	MEAN	48.652	49.613
	STD	17.046	18.529

Note: STA indicates the statistic; MIN indicates the minimum; MAX indicates the maximum; MEAN indicates the average, STD indicates the standard deviation; data are missing in 2011, 2013, and 2014.

**Table 4:** Results of the panel unit root tests at the different scales.

Variables	Provincial Scale			City Scale		
	LLC	ADF	PPS	LLC	ADF	PPS
CA	−2.79 ***	59.65	62.02	−10.99 ***	780.66 ***	824.36 ***
NP	10.64	20.57	9.13	1.68	555.78 ***	464.29 ***
LPI	−5.59 ***	128.58 ***	152.14 ***	−22.21 ***	1041.73 ***	1087.70 ***
LSI	−10.22 ***	108.60 ***	106.70 ***	−31.87 ***	1294.65 ***	1353.57 ***
ENN_MN	−11.39 ***	141.47 ***	135.21 ***	−2.93 ***	1379.38 ***	1479.34 ***
PLADJ	−12.51 ***	147.87 ***	147.79 ***	−39.98 ***	1590.01 ***	1716.48 ***
COHESION	−4.60 ***	89.94 ***	103.14 ***	−40.31 ***	1444.59 ***	1640.67 ***
*Population*	4.68	72.94 **	88.48 ***	−91.11 ***	903.95 ***	977.43 ***
*GDP*	6.23	21.86	32.94	23.96	250.40	255.33
PM_2.5_	−14.47 ***	205.54 ***	284.74 ***	−74.50 ***	2753.93 ***	3578.73 ***

Note: Significant at the ** 5% level, and *** 1% level. LLC represents the Levin, Lin, and Chu test [34]; ADF represents the ADF–Fisher chi-square test [43]; and PPS represents the PP-Fisher chi-square test [43].

**Table 5:** The relationships between the UF and PM_2.5_ concentration at the different scales.

Variable	Coefficient (Provincial Scale)		Coefficient (City Scale)	
	Fixed Effect	Random Effect	Fixed Effect	Random Effect
CA	0.000149 ***	−2.19 × 10^−5^	7.16 × 10^−5^ ***	5.71 × 10^−5^ **
NP	−0.033436 **	−0.112956 ***	−0.110307 *	−0.080818
LPI	−15.85242 ***	−7.337464	−0.377232 ***	−0.403132 ***
LSI	−2.106767 ***	2.545653 ***	−2.286485 ***	−2.150084 ***
ENN_MN	−2.44 × 10^−5^ ***	8.70 × 10^−5^ ***	−9.44 × 10^−5^ ***	−9.61 × 10^−5^ ***
PLADJ	−0.495721	0.654456 *	0.188681	0.124223
COHESION	0.727133 **	−0.797569 **	−0.041554	0.041326
*Population*	5.13 × 10^−5^	0.003239 ***	0.001401	0.000833
*GDP*	−6.96 × 10^−5^	0.000247 ***	2.96 × 10^−5^	6.30 × 10^−5^
R^2^	0.467900	0.389188	0.097361	0.031573
Adjusted R^2^	0.432427	0.372376	0.089557	0.028002
F-statistic	13.19020	23.15028	12.47607	8.842340
Probability (F-statistic)	0.000000	0.000000	0.000000	0.000000

Note: Significant at * 10% level, ** 5% level, and *** 1% level.

**Table 6:** The relationships between the UF and PM_2.5_ concentration in the different regions at the provincial scale.

Variable	Eastern Provinces		Central Provinces		Western Provinces	
	Fixed Effect	Random Effect	Fixed Effect	Random Effect	Fixed Effect	Random Effect
CA	−0.000104 ***	−8.70 × 10^−5^ ***	−0.000226 *	−0.000225 *	2.40 × 10^−5^	−5.36 × 10^−5^
NP	−0.164934 ***	−0.192234 ***	−0.001218 *	−0.001613 **	−0.152360 ***	−0.128675 ***
LPI	13.54699 *	9.878880 ***	90.60625 ***	80.68187 ***	12.26049	21.00478
LSI	9.744146 ***	9.632921 **	7.981017 **	7.219544 **	−1.946943	−0.694878
ENN_MN	−1.64 × 10^−5^	−1.90 × 10^−5^	−3.85 × 10^−5^	−0.000120	4.69 × 10^−5^ ***	5.01 × 10^−5^ ***
PLADJ	4.072971 ***	4.236901 ***	3.951412 ***	3.958976 ***	−0.652470	−0.499892
COHESION	−5.321512 ***	−5.188210 ***	−2.776398 ***	−2.657545 ***	1.440029 *	1.267429 *
*Population*	5.52 × 10^−5^	−1.63 × 10^−5^	0.003348 ***	0.003601 ***	−0.001530 *	−0.001618 **
*GDP*	0.000312 **	0.000252 **	7.30 × 10^−5^	−1.12 × 10^−5^	0.001672*	0.001992 ***
R^2^	0.912831	0.857158	0.901512	0.866379	0.509384	0.487694
Adjusted R^2^	0.890507	0.843481	0.871537	0.851533	0.423527	0.452764
F-statistic	40.89031	62.67433	30.07586	58.35489	5.932886	13.96203
Probability (F-statistic)	0.000000	0.000000	0.000000	0.000000	0.000000	0.000000

Note: Significant at the * 10% level, ** 5% level, and *** 1% level.

**Table 7:** The relationships between the UF and PM_2.5_ concentration in the different regions at the city scale.

Variable	Eastern Cities		Central Cities		Western Cities	
	Fixed Effect	Random Effect	Fixed Effect	Random Effect	Fixed Effect	Random Effect
CA	0.000107 ***	7.24 × 10^−5^ **	0.000152 ***	0.000119 *	−0.000173	−0.000363 **
NP	−1.116592 ***	−1.075780 ***	−0.307969 **	−0.237743	0.569279 ***	0.543614 ***
LPI	−0.564496 ***	−0.582432 ***	−1.083794 *	−0.927409 *	−3.817160 ***	−3.157423 ***
LSI	2.055063	3.001070 **	−1.340814 *	−1.251308	−8.881376 ***	−7.310335 ***
ENN_MN	−0.000106 **	−7.86 × 10^−5^	−0.000150 *	−0.000152 ***	−6.28 × 10^−5^ *	−7.96 × 10^−5^ **
PLADJ	−0.097256	−0.192349	0.319850 ***	0.284170 *	0.323929	0.259471
COHESION	0.102646	0.202534	−0.274463 **	−0.224378	0.479088	0.647384 **
*Population*	0.010097 ***	0.008177 ***	0.004254 **	0.003932 **	−0.006029 **	−0.007334 ***
*GDP*	5.34 × 10^−5^	6.69 × 10^−5^ **	1.02 × 10^−5^	1.34 × 10^−5^ **	2.14 × 10^−5^	4.59 × 10^−5^ ***
R^2^	0.103133	0.045942	0.137810	0.059979	0.213630	0.150234
Adjusted R^2^	0.086073	0.038248	0.127557	0.055221	0.172449	0.131716
F-statistic	6.045331	5.971130	13.44152	12.60530	5.187533	8.112916
Probability (F-statistic)	0.000000	0.000000	0.000000	0.000000	0.000000	0.000000

Note: Significant at the * 10% level, ** 5% level, and *** 1% level.

## Data Availability

Not applicable.

## References

[1] Huang Z., Wei Y.D., He C., Li H. (2015). Urban land expansion under economic transition in China: A multi-level modeling analysis. Habitat Int..

[2] Chen M., Liu W., Tao X. (2013). Evolution and assessment on China’s urbanization 1960–2010: Under-urbanization or over-urbanization?. Habitat Int..

[3] Zhao Y., Wang S., Aunan K., Seip H.M., Hao J. (2006). Air pollution and lung cancer risks in China—A meta-analysis. Sci. Total Environ..

[4] Alam M.S., Hyde B., Duffy P., McNabola A. (2018). Analysing the Co-Benefits of transport fleet and fuel policies in reducing PM_2.5_ and CO_2_ emissions. J. Clean. Prod..

[5] Chow J.C., Watson J.G., Lowenthal D.H., Solomon P.A., Magliano K.L., Ziman S.D., Richards L.W. (1993). PM_10_ and PM_2.5_ compositions in California’s San Joaquin Valley. Aerosol Sci..

[6] Pui D.Y., Chen S.-C., Zuo Z. (2014). PM_2.5_ in China: Measurements, sources, visibility and health effects, and mitigation. Particuology.

[7] Fan C., Tian L., Zhou L., Hou D., Song Y., Qiao X., Li J. (2018). Examining the impacts of urban form on air pollutant emissions: Evidence from China. J. Environ. Manag..

[8] Hailin W., Zhuang Y., Ying W., Yele S., Hui Y., Zhuang G., Zhengping H. (2008). Long-term monitoring and source apportionment of PM_2.5_/PM_10_ in Beijing, China. J. Environ. Sci..

[9] Ewing R.H., Pendall R., Chen D.D. (2002). Measuring Sprawl and Its Impact.

[10] Bereitschaft B., Debbage K. (2013). Urban form, air pollution, and CO_2_ emissions in large US metropolitan areas. Prof. Geogr..

[11] Yuan M., Huang Y., Shen H., Li T. (2018). Effects of urban form on haze pollution in China: Spatial regression analysis based on PM_2.5_ remote sensing data. Appl. Geogr..

[12] Zhao H., Guo S., Zhao H. (2019). Quantifying the impacts of economic progress, economic structure, urbanization process, and number of vehicles on PM_2.5_ concentration: A provincial panel data model analysis of China. Int. J. Environ. Res. Public Health.

[13] Liu Y., Wu J., Yu D., Ma Q. (2018). The relationship between urban form and air pollution depends on seasonality and city size. Environ. Sci. Pollut. Res..

[14] Tao Y., Zhang Z., Ou W., Guo J., Pueppke S.G. (2020). How does urban form influence PM_2.5_ concentrations: Insights from 350 different-sized cities in the rapidly urbanizing Yangtze River Delta region of China, 1998–2015. Cities.

[15] Liu Y., Arp H.P.H., Song X., Song Y. (2017). Research on the relationship between urban form and urban smog in China. Environ. Plan. B Urban Anal. City Sci..

[16] Shi K., Wang H., Yang Q., Wang L., Sun X., Li Y. (2019). Exploring the relationships between urban forms and fine particulate (PM_2.5_) concentration in China: A multi-perspective study. J. Clean. Prod..

[17] Shi K., Li Y., Chen Y., Li L., Huang C. (2019). How does the urban form- PM_2.5_ concentration relationship change seasonally in Chinese cities? A comparative analysis between national and urban agglomeration scales. J. Clean. Prod..

[18] Feng H., Zou B., Tang Y. (2017). Scale-and region-dependence in landscape- PM_2.5_ correlation: Implications for urban planning. Remote Sens..

[19] Ma Q., He C., Wu J. (2016). Behind the rapid expansion of urban impervious surfaces in China: Major influencing factors revealed by a hierarchical multiscale analysis. Land Use Policy.

[20] Parenteau M.-P., Sawada M.C. (2011). The modifiable areal unit problem (MAUP) in the relationship between exposure to NO_2_ and respiratory health. Int. J. Health Geogr..

[21] Arbia G., Petrarca F. (2011). Effects of MAUP on spatial econometric models. Lett. Spat. Resour. Sci..

[22] Fang C., Song J., Zhang Q., LI M. (2005). The formation, development and spatial heterogeneity patterns for the structures system of urban agglomerations in China. Acta Geogr. Sin. Chin. Ed..

[23] Chu H.-J., Huang B., Lin C.-Y. (2015). Modeling the spatio-temporal heterogeneity in the PM_10_-PM_2.5_ relationship. Atmos. Environ..

[24] Fang C., Wang S., Li G. (2015). Changing urban forms and carbon dioxide emissions in China: A case study of 30 provincial capital cities. Appl. Energy.

[25] Stone B. (2008). Urban sprawl and air quality in large US cities. J. Environ. Manag..

[26] He C., Liu Z., Tian J., Ma Q. (2014). Urban expansion dynamics and natural habitat loss in China: A multiscale landscape perspective. Glob. Chang. Biol..

[27] McGarigal K., Cushman S.A., Ene E. (2012). FRAGSTATS v4: Spatial Pattern Analysis Program for Categorical and Continuous Maps. Computer Software Program Produced by the Authors at the University of Massachusetts, Amherst. http://www.umass.edu/landeco/research/fragstats/fragstats.html.

[28] Van Donkelaar A., Martin R.V., Li C., Burnett R.T. (2019). Regional estimates of chemical composition of fine particulate matter using a combined geoscience-statistical method with information from satellites, models, and monitors. Environ. Sci. Technol..

[29] Van Donkelaar A., Martin R.V., Brauer M., Boys B.L. (2015). Global fine particulate matter concentrations from satellite for long-term exposure 2 assessment 3. Assessment.

[30] Rohde R.A., Muller R.A. (2015). Air pollution in China: Mapping of concentrations and sources. PloS ONE.

[31] Han L., Zhou W., Pickett S.T., Li W., Li L. (2016). An optimum city size? The scaling relationship for urban population and fine particulate (PM_2.5_) concentration. Environ. Pollut..

[32] Lin G., Fu J., Jiang D., Hu W., Dong D., Huang Y., Zhao M. (2014). Spatio-temporal variation of PM_2.5_ concentrations and their relationship with geographic and socioeconomic factors in China. Int. J. Environ. Res. Public Health.

[33] Shi K., Shen J., Wang L., Ma M., Cui Y. (2020). A multiscale analysis of the effect of urban expansion on PM_2.5_ concentrations in China: Evidence from multisource remote sensing and statistical data. Build. Environ..

[34] Levin A., Lin C.-F., Chu C.-S.J. (2002). Unit root tests in panel data: Asymptotic and finite-sample properties. J. Econom..

[35] Shi K., Chen Y., Yu B., Xu T., Chen Z., Liu R., Li L., Wu J. (2016). Modeling spatiotemporal CO_2_ (carbon dioxide) emission dynamics in China from DMSP-OLS nighttime stable light data using panel data analysis. Appl. Energy.

[36] Borenstein M., Hedges L.V., Higgins J.P., Rothstein H.R. (2010). A basic introduction to fixed-effect and random-effects models for meta-analysis. Res. Synth. Methods.

[37] Wen H., Tao Y. (2015). Polycentric urban structure and housing price in the transitional China: Evidence from Hangzhou. Habitat Int..

[38] Bailliu J., Kruger M., Toktamyssov A., Welbourn W. (2019). How fast can China grow? The Middle Kingdom’s prospects to 2030. Pac. Econ. Rev..

[39] Hu H. (1935). The distribution of population in China. Acta Geogr. Sin..

[40] Zhou L., Zhou C., Yang F., Che L., Wang B., Sun D. (2019). Spatio-temporal evolution and the influencing factors of PM_2.5_ in China between 2000 and 2015. J. Geogr. Sci..

[41] Yan D., Lei Y., Shi Y., Zhu Q., Li L., Zhang Z. (2018). Evolution of the spatiotemporal pattern of PM_2.5_ concentrations in China–A case study from the Beijing-Tianjin-Hebei region. Atmos. Environ..

[42] Wang S., Zhou C., Wang Z., Feng K., Hubacek K. (2017). The characteristics and drivers of fine particulate matter (PM_2.5_) distribution in China. J. Clean. Prod..

[43] Maddala G.S., Wu S. (1999). A comparative study of unit root tests with panel data and a new simple test. Oxf. Bull. Econ. Stat..

[44] Gan C.-H., Zheng R.-G. (2009). An empirical study on change of industrial structure and productivity growth since the reform and opening-up—A test for the structure-bonus hypotheses from 1978 to 2007 in China. China Ind. Econ..

[45] Wang Z., Jia H., Xu T., Xu C. (2018). Manufacturing industrial structure and pollutant emission: An empirical study of China. J. Clean. Prod..

[46] Wolfe D.A. (2010). Economy; Society, The strategic management of core cities: Path dependence and economic adjustment in resilient regions. Camb. J. Reg. Econ. Soc..

[47] Ou J., Liu X., Li X., Chen Y. (2013). Quantifying the relationship between urban forms and carbon emissions using panel data analysis. Landsc. Ecol..

[48] Tan M. (2007). Climatic differences and similarities between Indian and East Asian Monsoon regions of China over the last millennium: A perspective based mainly on stalagmite records. Int. J. Speleol..

[49] Jin Q., Fang X., Wen B., Shan A. (2017). Spatio-temporal variations of PM_2.5_ emission in China from 2005 to 2014. Chemosphere.

[50] Guan Q., Cai A., Wang F., Yang L., Xu C., Liu Z. (2017). Spatio-temporal variability of particulate matter in the key part of Gansu Province, Western China. Environ. Pollut..

[51] Chen Y., Li X., Zheng Y., Guan Y., Liu X. (2011). Estimating the relationship between urban forms and energy consumption: A case study in the Pearl River Delta, 2005–2008. Landsc. Urban Plan..

[52] Doronzo D.M., de Tullio M.D., Pascazio G., Dellino P., Liu G. (2015). On the interaction between shear dusty currents and buildings in vertical collapse: Theoretical aspects, experimental observations, and 3D numerical simulation. J. Volcanol. Geotherm. Res..

